# Elderly women living alone in Spain: the importance of having children

**DOI:** 10.1007/s10433-017-0415-6

**Published:** 2017-02-17

**Authors:** David Reher, Miguel Requena

**Affiliations:** 1 Universidad Complutense de Madrid, Madrid, Spain; 2 Universidad Nacional de Educacion a Distancia, Madrid, Spain

**Keywords:** Living alone, Completed fertility, Childlessness, Family support, Vulnerability, Spain

## Abstract

Our goal in this paper is to analyse the extent to which completed fertility, and in particular childlessness, is a valid predictor of living alone at advanced ages, an increasingly important residential option in advanced societies with crucial implications for social policy design and the organization of welfare services. Based on micro-data from the 2011 Spanish population census, logistic regression techniques are used to assess the impact of fertility on living alone among elderly women net the effect of age, marital status, educational attainment, and other standard population controls. Our results show a clear relationship between completed fertility and living alone. Childlessness is strongly associated with living alone, while having offspring acts as a powerful buffer against living alone, particularly in larger families. A relevant conclusion of this study is that a growing deficit of family resources available for the elderly women will take place in those societies where low fertility and high rates of childlessness have prevailed in recent decades, leading to substantial growth in the number of childless elderly women and in the incidence of living alone during later life.

## Introduction

The prevalence of living alone during later life varies widely across developed countries, but everywhere its growth has been remarkable in recent decades, even in societies with traditionally strong family ties. At the same time, in many of these developed countries fertility has been very low for decades now, a pattern that is especially visible in societies with robust family systems located mainly in the Eastern and Southern fringes of Europe. These trends have led to simultaneous increases in living alone and in childlessness. Comparative studies have brought to light that childless elders are more likely than parents to live alone and have suggested that there is a causal link between both phenomena (Koropecckyj-Cox and Call 2007). This connection between low fertility and living alone warrants further analysis not only because of the importance of single living among older people but also because any link to the reproductive characteristics of any given society will enable us to forecast future trends based on current fertility.

According to Burch and Matthews (1987), household status can be considered as a composite good with which individuals seek to satisfy several goals—privacy, companionship, domestic services, personal care, and consumption economies of scale. Living arrangements are a means for achieving several ends whose specific combination is subject to the tradeoffs confronted by individuals deciding with whom to live. The particular mixture of domestic goods chosen by household members is the result of preferences towards their different ingredients, changing economic and social–psychological cost, and available resources required to obtain them. Along this line, in developed societies it has been suggested that the demand for these domestic goods depends on preferences for autonomy and privacy, income levels, and the availability of kin. In turn, past demographic trends—and above all fertility (Kobrin 1976; Bongaarts 1983; Wister and Burch 1983)—are crucial determinants of the supply of kin with whom to live. From this perspective, living arrangements are necessarily limited by family size.

Other things being equal, rising income, growing preferences for autonomy, improved health, and reduced pools of kin can be expected to promote residential independence among elderly people. During the last four decades, a consistent body of research (Kobrin 1976; Pample 1983; Wister and Burch 1983; Macunovich et al. 1995; Wolf 1995; Iacovou 2000; Gaymu et al. 2006; Koropecckyj-Cox and Call 2007) has shown that older female co-residential options, and in particular living alone, tend to be shaped by an individual’s past fertility. Most of this literature refers to non-familistic societies and deals with the relatively recent past. The extent to which current increase in single living among older women is related to the availability of children, especially in a familistic society like Spain characterized by traditionally strong preferences for co-residence with kin (Reher 1998), is unquestionably relevant.

On the other hand, under the assumption that behaviour in the earlier phases of the life course has consequences at older ages, more recent research has aptly considered childlessness as ‘a road less taken’ (Dykstra and Hagestad 2007), clarifying the different ways in which not having children may structure people’s lives in macro-, meso-, and micro-level contexts (Dykstra 2009). With respect to its structural influence at the micro-level—and despite the important work done in the field on late-life psychological, economic, and social outcomes of childlessness (Dykstra 2015; Ivanova and Dykstra 2015)—we feel that the possibility of living alone as a delayed effect of being childless deserves further attention. This is especially the case in a strong family-oriented society such as Spain’s in which kin interventions are generally considered an important part of the well-being of older people. In these scenarios, not having children plus living alone would inevitably lead to a shortage of the type of kin support and contact normally considered important for quality ageing (Lowenstein et al. 2003; Tomassini et al. 2004; Puga et al. 2006).

Women constitute an appropriate subpopulation to analyse living alone and fertility jointly. Their longevity is greater in both relative and absolute terms than among men. Moreover, tracking reproductive histories is far easier for women. In Spain, the proportion of women 65+ living alone has increased by 43 % since 1981, reaching an unprecedented 28% of all older women (1279,486 persons) in 2011. Despite the familistic character of Spanish society, autonomy is a rising value among older people (López-Doblas 2005). Consequently an increase in the proportions of elderly people living alone has been observed in recent decades in the country, along with a decrease in those living with their own children or with other people (Pérez Ortiz 2006; Abellán et al. 2007; Zueras and Miret 2013). At the same time, fertility declined sharply in Spain towards the end of the 1970s and has remained extremely low since, leading to sharp increases in the levels of childlessness and decreasing sibset size among cohorts born in the 1950s and 1960s. In 2011, 16% (725,642) of all women 65+ in Spain were childless (Esteve et al. 2016), with substantially higher levels (>20–22%) for women who were between 50 and 65 years of age in the same 2011 census. Based on past demographic trends, the number of childless older people can be expected to increase sharply in the coming decades (Esteve et al. 2016).

In contemporary societies, the existence of parallel upward trends in low fertility and living alone during later life is important for a number of reasons. First, small social networks associated with reduced fertility and childlessness (Koropeckyj-Cox 1998; Dykstra and Wagner 2007), and the social isolation that tends to come with solo living is known to be significantly associated with mortality (all causes) and with an ample range of diseases (Umberson and Montez 2010; Steptoe et al. 2013; Cacioppo et al. 2014). Living alone has been shown to be directly linked to higher levels of disease and disability (Kharicha et al. 2007). Second, while recent research has shown mixed evidence regarding the impact of fertility on the psychological well-being of older women (Zhang and Hayward 2001; Kohler et al. 2005; Hansen et al. 2009; Gibney 2012; Kravdal 2014; Gibney et al. 2017), there is little doubt that living alone reduces satisfaction with life and perceived quality of life in old age (Jakobsson et al. 2004; Gaymou and Springer 2012). Third, even though reduced fertility does not necessarily imply a large deficit of social support, except in cases of poor health (Bachrach 1980; Albertini and Mencarini 2014), living alone lowers the levels of social and familial support at older ages (Thompson and Krause 1998; Aykan 2003; Chen et al. 2014). In sum, childless women who live alone are a potentially vulnerable group that is at risk of having insufficient support during old age (Larsson and Silverstein 2004).

In this paper, we make use of the 2011 Spanish population census to assess the extent to which completed fertility is a valid predictor of living alone among women aged 65+. If our basic hypothesis that fertility is a relevant determinant of living alone can be validated, we will be in a position to forecast an important part of future trends in solo living among older women based on recent patterns of fertility and, in particular, childlessness. The linked occurrence of low fertility and living alone affecting different stages of the life span will have crucial implications for social policy design, placing a heavy burden on the organization of welfare services in advanced societies, especially in strong family ones where up to now a substantial part of the support for older people has come mainly from kin.

## Living arrangements of older women in Spain

**Fig. 1:**
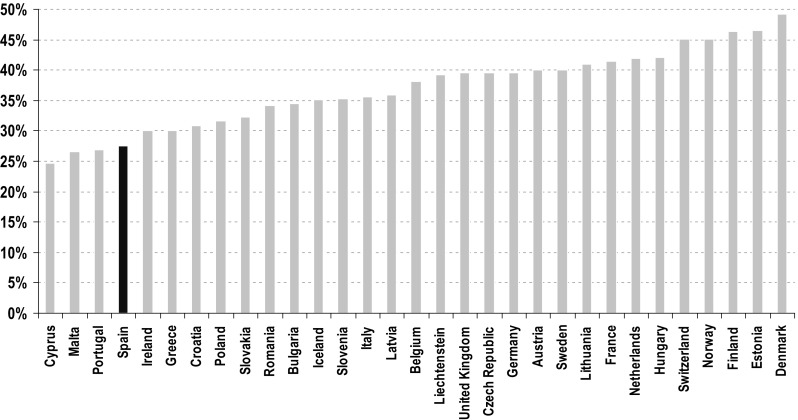
Women 65+ living alone in Europe, 2011. *Source*: Eurostat

Ample differences can be observed in the prevalence of older women living alone in developed societies. Figure 1 shows the proportions of women 65+ living alone in 31 European countries in 2011. Levels vary widely, from a maximum of 45% or more in some Nordic (Norway, Finland, and Denmark) and Baltic (Estonia) countries and Switzerland, to a minimum of about 25% in a number of Mediterranean and Southern countries (Cyprus, Malta, Portugal, and Spain). Slightly higher levels of living alone (about 30%) are found in several Eastern countries (Croatia, Poland, Slovakia, Romania, and Bulgaria) as well as Latvia, Ireland, Greece, and Italy. Another group, with levels around 40% is composed of central continental countries (Belgium, Germany, Austria, France), the UK, Czech Republic, Sweden, Lithuania, and Hungary.

These differences in national proportions of older women living alone are determined primarily by several demographic compositional factors such as age, sex, marital status, education, or completed fertility (Gaymu et al. 2006). In addition, certain cultural elements also play a role. In particular, the observed variation in living arrangements in these European countries can be interpreted in terms of the intensity of their underlying levels of familism (Reher 1998) and appear to fit into a geographical continuum ranging from North and West to South and East (Iacovou and Skew 2011). Although the grouping of countries into distinct categories is far from perfect, the data from the 2011 round of European population censuses confirm the existence of this rather robust family continuum: where the strength of family ties, networks and practices is greater, the proportion of elderly women living alone tends to be lower. As a previous study (Zueras and Miret 2013) has shown, in Europe the country differences in the propensity of older people to live alone persist even after controlling for several structural variables (gender, age, marital status, level of education, and employment status) and they mirror the North/South familistic axis.

Beyond the persistent North/South distribution in levels of living alone among older women, the most relevant aspect of the data presented here is that everywhere levels of non-family living arrangements among older people are increasing, even—or perhaps especially so—in familistic Southern Europe. The Spanish case is very clear on this point. Despite being a society with strong family ties and kinship networks, living alone has risen substantially among all subpopulations, including older women (Requena 2004; Zueras and Miret 2013). As given in Table 1, over the past three decades single living has been steadily increasing among women 65+. In fact, the living arrangement exhibiting the largest growth in absolute numbers during this period is precisely single living (+801,567 persons). Obviously, an important part of this increase is accounted for by the overall growth of the older female population (+2207,211 persons). Despite this, the number of women living alone increased much more in relative terms than the extent of the older female population as a whole (growth ratios of 2.68 and 1.88, respectively). It is also true that in relative terms the growth of the number of women in institutions has been greater (ratio 2011/1981 = 2.92) than that of women living alone (2.68), though institutionalized populations continue to represent a limited option for older women (4.3% of all these women in 2011 versus 27% living alone). Among older women in Spain, the only household structure whose relative growth competes with solo living is that of married women living with children (2.36), though once again this is a type of living arrangement observed in one out of ten (9.8%) of Spanish women 65+ in 2011. Another characteristic of this period is the increase in older women continuing to live with a partner (usually a husband), the result of improving health among adult men as well as the fact that the incidence of divorce was still relatively low in a traditional country such as Spain.

**Table 1:** Living arrangements of Spanish women 65+. *Source*: 1981, 1991, 2001 and 2011 Spanish Population Censuses

					∆ 1981–2011	
	1981	1991	2001	2011	2011–1981	2011/1981
All women 65+	25,12,798	31,61,601	40,27,953	47,20,009	22,07,211	1.88
Living alone	477,919	6,96,910	10,43,471	12,79,486	8,01,567	2.68
With partner only	6,82,722	7,86,718	10,49,939	14,07,915	7,25,193	2.06
With children only	2,43,627	2,48,488	3,16,430	4,71,469	2,27,842	1.94
With partner and children	1,96,259	2,55,737	3,96,975	4,63,027	2,66,768	2.36
Others arrangements	8,43,395	10,73,615	11,05,531	8,97,220	53,825	1.06
In institutions	68,876	1,00,133	1,15,607	2,00,892	1,32,016	2.92
Living alone	19.0%	22.0%	25.9%	27.1%	8.1%	1.43
With partner only	27.2%	24.9%	26.1%	29.8%	2.7%	1.10
With children only	9.7%	7.9%	7.9%	10.0%	0.3%	1.03
With partner and children	7.8%	8.1%	9.9%	9.8%	2.0%	1.26
Others arrangements	33.6%	34.0%	27.4%	19.0%	−14.6%	0.57
In institutions	2.7%	3.2%	2.9%	4.3%	1.5%	1.55

The result of these changes in living arrangements is that more than one of the four older women (27%) lived alone in Spain in 2011, while thirty years earlier less than one of five (19%) did. Solo living was the second most popular residential alternative among elderly Spanish women in 2011, just below women living with a partner (30%). As is happening in other familistic and non-familistic societies, living alone is becoming an increasingly preferred alternative that is likely to continue to increase in the near future. It thus deserves special attention.

## Method

### Data used

Our analysis is based on micro-data from the 2011 Spanish population and housing census and, in particular, on a broad random sample (≈10% of the universe) of the women 65+ made up of 472,937 observations representing a total of 4519,118 persons. The large sample size guarantees very small sampling errors and allows for high confidence levels in estimates (α = 0.001). The 2011 Spanish census gathers data on several socio-demographic and household characteristics and also records the number of children ever born for women (but not for men). The fact that Spain and Ireland are the only developed nations to continue to include information for this variable in the most recent round of censuses increases the interest of this study because it offers detailed and up-to-date information about this important issue.

Although population censuses do not constitute databases designed specifically for studying fertility, their use for this purpose is both possible and profitable. Tracing the number of children ever born to women with completed fertility by means of a single census is an interesting opportunity (David and Sanderson 1990) that has been already exploited in a number of occasions (Requena and Salazar 2006; 2014; Reher and Requena 2015a, b). However, several precautions should be taken. Since fertility data on censuses may reflect potential biases due to the selective effects of mortality^1^ and migration, problems of coverage, the tendency of informants to misreport data increasingly with age, and the possibility of inaccurately reporting childlessness, some kind of quality check is required. Following Feeney (1995), estimates of completed fertility and in particular of childlessness for the same cohorts in different censuses have been compared as a measure to control data quality. Despite the fact that the census micro-data only include women living in households and therefore exclude institutionalized populations, after implementing several different controls, the results can be considered satisfactory yielding acceptable estimates for childlessness.

### Variables

Our dependent variable is based on the living arrangements of older women coded as a straightforward dichotomous variable: living alone and living with others. Our main independent variable is children ever born (coded into four categories: 0, 1/2, 3, and 4 +).^2^ In the models used in this paper, other key covariates were included: age (coded into six five-year groups from 65–69 to 90 +), current marital status ([4]: never married, married, separated/divorced, and widowed), and educational attainment ([5]: less than primary, primary, low secondary, upper secondary, and university). Region of residence ([5]: North, North Centre, Centre, East, and South), rural/urban divide linked to city size (into 5 ordered categories), migratory status (native versus immigrants), and type of housing ([3]: home owner, rented and other forms) have been also added to the models as population controls.

### Statistical analysis

Since the object of analysis (living alone versus living with others) is intrinsically binary, logistic regression is the most appropriate statistical technique. We estimate three logistic regression models sequentially adjusting for different socio-demographic characteristics: first, age, marital status, and completed fertility enter the model; next, education (our best proxy for socio-economic status) is added; and finally, additional controls for the remaining covariates (region of residence, rural/urban divide, migratory status, and type of housing) are included in the full pooled model. The goal here is to check whether the expected association between completed fertility and living alone holds after the population controls are taken into account within the pooled regression model. Following this hierarchical strategy, we assess both the impact on living alone of being childless as opposed to being a mother and the effect of the number of children ever born on the likelihood of living alone among women who have had children. For the sake of parsimony, only the full pooled model is shown here. (The other two models are available upon request). The model presents odds ratios with robust standard errors, *p* values, and 99.9% confidence intervals for all variables.

## Results

### Descriptive

Table 2 presents descriptive statistics on living arrangements of Spanish women aged 65+ in 2011. Bivariate results reveal: (1) the existence of a nonlinear relationship between age and living alone, with proportions of women living alone increasing up to 85–89 years of age and then decreasing; (2) a disproportionate association of living alone with the status of not being married (single, separated or divorced, and widow women); and (3) a u-shaped relation with education, with higher levels of solo living observed among less and more educated women. The results for the rest of the variables included in the analysis conform to expectations: a high prevalence of living alone among women born in Spain, living in rental housing, and residing in big cities. Region of residence does not show relevant geographical differences, except for a slightly higher prevalence of living alone among women in the Northern part of the country. Finally, proportions of women living alone decrease steadily with the number of children ever born.

**Table 2:** Descriptive statistics of Spanish women 65+ by living arrangements. *Source*: 2011 Spanish Census. Data weighted by sample weights

	Living with others	Living alone	N
All women 65+	71.7%	28.3%	45,19,118
*Age*			
65–69	82.1%	17.9%	11,36,089
70–74	76.7%	23.3%	9,25,968
75–79	69.1%	30.9%	10,23,865
80–84	62.1%	37.9%	7,69,902
85–89	59.6%	40.4%	4,48,269
90+	67.1%	32.9%	2,15,025
*Marital status*			
Single	53.0%	47.0%	3,43,703
Married	98.2%	1.8%	21,35,803
Sep/div	47.8%	52.2%	1,34,033
Widow	47.0%	53.0%	19,05,579
*Education*			
Less than primary	70.8%	29.2%	18,40,200
Primary	71.4%	28.6%	13,14,386
Lower secondary	74.6%	25.4%	8,46,349
Upper secondary	71.7%	28.3%	2,84,488
University	69.8%	30.2%	2,33,695
*Housing tenancy*			
Ownership	72.1%	27.9%	38,81,857
Rented	63.8%	36.2%	2,87,426
Other	73.7%	26.3%	3,49,834
*Rural–Urban divide*			
2000–	74.9%	25.1%	4,04,910
2001–5000	75.3%	24.7%	3,45,232
5001–10000	74.8%	25.2%	3,51,898
10001–20000	75.1%	24.9%	4,44,311
20001–100000	71.9%	28.1%	4,09,580
100001–500000	71.1%	28.9%	13,28,332
500000+	68.1%	31.9%	12,34,854
*Migratory status*			
Native	71.3%	28.7%	43,33,411
Immigrant	79.6%	20.4%	1,85,707
*Region*			
North West	74.6%	25.4%	8,08,298
Centre north	71.0%	29.0%	5,58,586
Centre	70.5%	29.5%	7,65,514
East	70.2%	29.8%	13,86,048
South	72.8%	27.2%	10,00,672
*Children ever born*			
0	61.7%	38.3%	7,25,642
1	71.3%	28.7%	4,86,819
2	73.5%	26.5%	13,10,184
3	74.2%	25.8%	9,56,420
4	74.3%	25.7%	5,30,325
5+	74.1%	25.9%	5,09,728
$${\text{Mean\;Children\;Ever\;Born}}\quad \frac{\text{Living\;with\;others}}{2.555}\frac{\text{Living\;alone}}{2.273}\;\frac{{{\text{Diff}} .}}{{\begin{array}{*{20}c} {0.282***} \\ \end{array} }}$$ $${\text{Average\;Household\;Size}}\quad \frac{\text{With\;children}}{2.238}\frac{\text{Childless}}{1.993}\;\frac{{{\text{Diff}} .}}{0.245***}$$			

* *p* < 0.05; ** *p* < 0.01; *** *p* < 0.001

In addition to these basic distributions, two summaries of the expected association between completed fertility and living arrangements are provided in the lower panel of the table: (1) the mean number of children ever born for women 65+ living with others is 2.55, yielding a statistically significant (*p* < 0.001) difference of +0.28 in comparison with women living alone and (2) the average household size of women having had children is 2.24 while only 1.99 persons on average reside in the households where childless women live (difference of –0.25, *p* < 0.001). In brief, the lower the number of children ever born, the higher the chances of living in a smaller household.

### Multivariate

Table 3 presents estimates of a logistic regression model for the probability of living alone among Spanish women 65+. The odds ratios in the table gauge the likelihood for these women of living alone in comparison with living with others while controlling for different covariates. The multivariate results tend to confirm the bivariate ones with regard to age (a quadratic association, but with the point of inflection placed in the age group 80–84) and marital status (very strong association with the status of being single and, above all, with being divorced or widowed). However, the inverted u-shaped relationship between educational attainment and living alone that was observed in the bivariate results disappears. Women with no formal education are less likely to live alone and, while there is no statistically significant difference in the likelihood of single living between women with primary, lower secondary, and upper secondary education (see the overlapping confidence intervals corresponding to the OR for these variables), a higher odds among more educated women can also be observed.

**Table 3:** Odds ratios of living alone of Spanish women 65+. *Source*: 2011 Spanish Census

	OR	SE	*p* > z	[99.9% conf. interval]	
*Age*					
65–69 [Reference]					
70–74	1.18	0.02	0.000	1.11	1.25
75–79	1.28	0.02	0.000	1.21	1.35
80–84	1.17	0.02	0.000	1.11	1.24
85–90	0.91	0.02	0.000	0.86	0.97
90+	0.54	0.01	0.000	0.50	0.58
*Marital status*					
Single [Reference]					
Married	0.03	0.00	0.000	0.03	0.03
Sep/div	1.96	0.06	0.000	1.77	2.17
Widow	2.20	0.05	0.000	2.05	2.36
*Children ever born*					
0 [Reference]					
1/2	0.63	0.01	0.000	0.59	0.67
3	0.58	0.01	0.000	0.54	0.62
4+	0.44	0.01	0.000	0.42	0.47
*Education*					
Less than primary [Reference]					
Primary	1.19	0.01	0.000	1.14	1.23
Lower secondary	1.14	0.02	0.000	1.08	1.20
Upper secondary	1.20	0.03	0.000	1.11	1.30
University	1.33	0.03	0.000	1.22	1.44
*Housing tenancy*					
Ownership [Reference]					
Rented	1.05	0.02	0.020	0.98	1.12
Other	0.66	0.01	0.000	0.62	0.70
*Rural–urban divide*					
2000– [Reference]					
2001–5000	0.98	0.02	0.263	0.92	1.04
5001–10000	1.00	0.02	0.858	0.94	1.07
10001–20000	0.99	0.02	0.707	0.93	1.06
20001–100000	1.10	0.02	0.000	1.03	1.19
100001–500000	1.17	0.02	0.000	1.12	1.23
500000+	1.17	0.02	0.000	1.12	1.23
*Migratory status*					
Native [Reference]					
Immigrant	0.53	0.02	0.000	0.47	0.60
*Region*					
North West [Reference]					
Centre North	1.48	0.03	0.000	1.40	1.58
Centre	1.44	0.03	0.000	1.36	1.53
East	1.45	0.02	0.000	1.37	1.53
South	1.24	0.02	0.000	1.17	1.31

As expected, a woman’s fertility history is strongly associated with living alone during old age, and this association is statistically significant even when adjusted for the population controls included in our model. Having offspring acts as a powerful buffer against living alone at later ages (see Table 3): having had one or two children versus remaining childless reduces the OR of living alone by a factor of 0.63 (99.9% confidence interval; CI 0.59–0.67); among women with 3 children the OR of living alone diminishes by a factor of 0.58 with regard to childless women (99.9% confidence interval; CI 0.54–0.62); and among women with 4+ children, the OR diminish by a factor of 0.44 (99.9% confidence interval; CI 0.42–0.47). Therefore, the powerful effect of childlessness on living alone is fully verified: our results show that childless women are not only more likely to live alone, but also that the odds of living alone decline as the number of children increases, a pattern especially visible among women with relatively higher numbers of children.

**Fig. 2:**
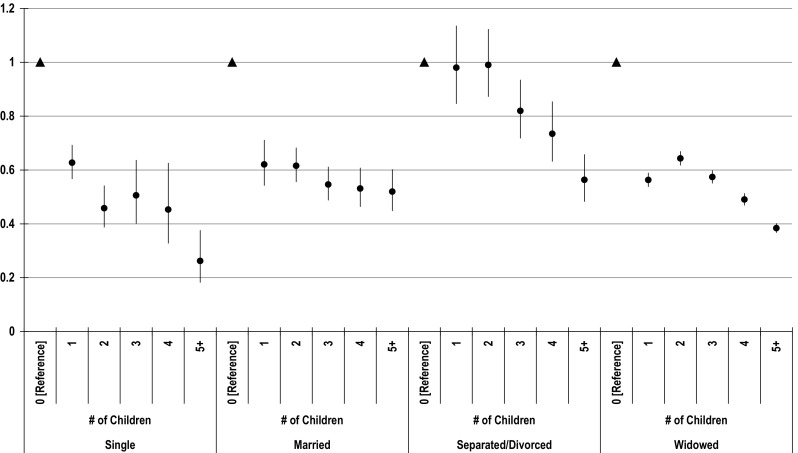
Adjusted odds ratios for living alone by marital status and children ever born, estimated from four separated models for each marital status. *Source*: 2011 Spanish Population Census. Author’s own elaboration

Since the fertility trajectories of these female cohorts developed in a social context ruled by traditional Catholic values where childbearing was for the most part restricted to married couples, the possibility of strong interactions between marital status and reproductive behaviour cannot not be discarded. In order to disentangle this interaction, different regression models for each marital status have been estimated. The adjusted odds ratios for living alone estimated from these four models confirm clearly that having children reduces the likelihood of living alone among women 65+ in every marital status considered (Fig. 2), thus leading to the conclusion that past fertility of older Spanish women affected living arrangements independent of marital status.

## Discussion

Up until now, research on the relationship between fertility and lifetime childlessness and living arrangements has been relatively scarce for developed countries. The goal of this report was to consider the case of Spain, a paradigmatic strong family society (Reher 1998) with a record of the lowest low fertility over the past forty years. Our basic hypothesis that childlessness is a relevant determinant of living arrangements among elderly women was fully confirmed for Spain with data based on a broad sample of the 2011 population census. A statistically significant and strong association was shown to exist between completed fertility and living alone. The number of children ever born among fertile women also proved to be a good predictor of living alone among older women, especially among women with larger sibsets. Since our results were adjusted for basic demographic and social characteristics, several potential confounders have been discarded.

An interesting aspect of this relationship between fertility and living alone is the extent to which the sex of surviving children modifies the co-residential options of elderly women. The pertinent literature (Lee 1992; IMSERSO 2014) has emphasized the importance of daughters as family caregivers, suggesting that the chances of living with a daughter are higher than living with a son. As the census database does not contain the sex of children ever born, including this variable in our models was not possible. However, observing the proportions in the census of older women living only with a daughter or a son was feasible and provided an indirect way of addressing this issue. Here the results were mixed. While the census data did not confirm the existence of an overall preference for older mothers to live with daughters rather than sons, it did reveal a distinctive pattern associated with age whereby the older the mother living only with one child, the higher the probability of living with a daughter. This finding corroborates that daughters are the main family caregivers as mothers age and become increasingly unable to take care of themselves. This only becomes clear among women >80 years of age, while at younger ages when they are far more capable of taking care of themselves, co-residence with a sole child tends to be co-residence with sons. In other words, as women enter into what has been called the ‘fourth age’, they certainly have a greater stake in having daughters than in having sons.

Part of the explanation for the basic association between fertility and living alone is strictly demographic: non-existent or small numbers of offspring shrink the pool of kin (children and grandchildren) available for co-residence, thus reducing the opportunities for living with others independent of any residential preferences. Furthermore, since low fertility and childlessness is most common among never married women, living alone during later life is also the result of having no partner. For an ample portion of elderly women, it is the lack of kin caused by childlessness that leads to single living. Not surprisingly and related to these results, at least in Spain, childless older people who do not live alone are also more likely than women with children to live in households with no kin at all (results not shown here). In addition, even among women who have had children the likelihood of living alone diminishes with the number of children even born. In sum, women’s marital and reproductive trajectories alter the opportunity structure for co-residence, shaping their living arrangements and ultimately fostering living alone during old age.

This straightforward demographic explanation for living alone in later life is not entirely satisfactory on three counts. (1) In the case of women with large families, there may be, in addition to the demographic mechanism, an element of educated values affecting family solidarity that is stronger in larger families than in smaller ones. (2) In the case of the 25% of older Spanish women with children who also live alone or the 62% of childless women who live with others, factors related to choice may also be relevant. There is an increasing preference for residential autonomy involved for all older women and most visibly for those with children who live alone or those who are childless and who live with others. (3) The ongoing improvements in the health and economic status of older people make living alone increasingly possible among them at increasingly older ages.

Census micro-data are of little help when examining preferences and do little to distinguish voluntary from involuntary behaviours. Nonetheless, the observed trends allow some room for interpretation. The growth of living alone over the past three decades among baby boom female cohorts—with historically low rates of childlessness—points to the existence of increases both in the preferences for solo living (López-Doblas 2005) and in the social and economic resources available for maintaining this kind of household (Zueras and Miret 2013). At the same time, the nonlinear effect of age on living alone among elderly Spanish women—first increasing then decreasing their rate of single living as they age—also indicates that the voluntary option for this type of living arrangement has its limits due to a decreasing ability to live alone that makes single living an increasingly difficult option beyond a certain age. It is at these very advanced ages, often called the ‘fourth age’, when childlessness becomes a major issue for these oldest old women who require support during this final phase of their lives.

### Implications for the future

Our results not only underscore the importance of offspring as a buffer against living alone during old age, but also suggest that there will be increasing shortfalls in family resources available for elderly women in the future. In societies where the fertility patterns prevailing during the second half of the twentieth century have included high proportions of small families with few or no offspring, a growing number of childless older women will face increasing shortages of kin and will be obliged to live alone in the coming decades, independent of their residential preferences. Along this same line, the sharp reduction in completed family size taking place over the past 40 years is undermining the weight of relatively large families (3 or more children) and limiting their ability to act as a buffer against living alone. The implications of shortages of immediate kin may be more difficult to manage in familistic societies like Spain where relatively low public support from welfare state institutions is compensated, at least in part, by the higher assistance, backing, help and aid coming from cohesive family networks (Puga et al. 2006; IMSERSO 2009). Since living arrangements constitute one of the most basic social resources available for older people providing them with proximate potential caregivers in case of impairment or illness, women living alone, be they childless or those with small sibsets, are likely to be a vulnerable group, particularly during later stages of their lives. In a country like Spain, these supportive living arrangements tend to be overwhelmingly family-based and the role of available kin in these circumstances should not be underestimated.

**Fig. 3:**
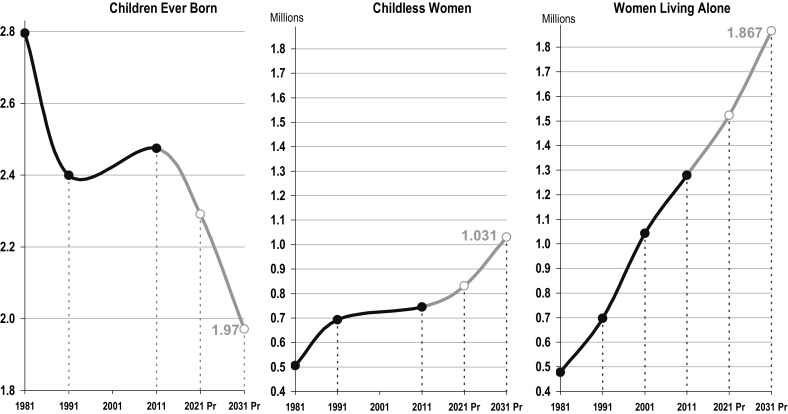
Spanish Women 65+. Fertility and living arrangements projection. *Source*: 1981, 1991, 2001 and 2011 Spanish Population Censuses and Population Projection (INE)

Although certainly not alone in the developed world, Spain represents an extreme example of these patterns due to its persistently low levels of fertility in recent decades. Projecting the completed fertility of women 65+ for the next two decades is a straightforward exercise given that women 45+ had ended their reproductive lives in 2011 and are unlikely to have any more children. Results here are conclusive (Fig. 3). In the next two decades, as the first cohorts of women participating in the baby bust become elderly themselves, the completed fertility of older women will begin to decrease sharply and the number of childless women will increase by a factor of 1.38 reaching an unprecedented total of 1031 million childless elderly women by 2031. Projecting the growth of childless women over the next twenty years among cohorts with completed fertility in 2011, and keeping their rates of living alone constant—a very conservative assumption—the estimated number of older women living alone in Spain will reach the unprecedented figure of almost 1.9 million within the next 15 years.

This revolution in single living will be tempered in part by the fact that in Spain the proportion of older women living with a partner will also increase in the near future, due to ongoing improvements in male survival (Abellán and Pujol 2013). Nevertheless, the growth of women living alone is perfectly compatible with this underlying counter trend, much as shown during the 1981–2011 period (see Table 1) when the growth of women living alone was much higher than that of women living with a partner. Moreover, while the increase in older women living with a partner is likely to persist in the future, it will do so at a diminished rate given the increasing importance of divorce among adult and elderly women.

We have chosen not to take this forecasting exercise beyond 2031, but alternative projections taking into account other components of these subpopulations (for instance, educational attainment) would point to even larger numbers of elderly women living alone in the next decades. If we push these projections a bit further to include women whose cohort fertility is well below replacement and levels of childlessness well above 20%, the number of childless older people and consequently the number of those living alone promise to increase until well past 2040.

In conclusion, the association between fertility and living alone has proved to be strong among older women in Spain. In particular, since both childlessness and single living are on the rise in most developed societies, this association should be considered a relevant aspect of ageing. Childlessness can and should be considered a crucial component for projections of the future numbers of elderly women living alone. It is important to test for this relationship in both familistic and non-familistic societies in order to ascertain the extent to which it is generalizable to other parts of the developed world and how it might vary in different contexts. If our general thesis is confirmed elsewhere, the long-term social implications of childlessness and low fertility will be far greater than what has normally been thought. It promises to become a key element not only in explaining present and future trends in living alone during later life but also in defining the limits of vulnerability during that crucial period of life.

## Footnotes

1 If we consider that the mean age of these women’s children is approximately 50 and base our estimates on the Human Mortality Database (2016) life tables for both sexes, it can be concluded that between 4 and 6 % of children born to older women present in the 2011 census would have died before the census was taken.

2 A model comparing childless women directly with women having had children independent of their number has been specified but is not included in the present paper. This model is available from the authors upon request.
